# Provider Perspectives on Barriers and Facilitators to Postpartum Care for Low-Income Individuals

**DOI:** 10.1089/whr.2021.0009

**Published:** 2021-07-16

**Authors:** Rachel S. Ruderman, Emma C. Dahl, Brittney R. Williams, Ka'Derricka Davis, Joe M. Feinglass, William A. Grobman, Michelle A. Kominiarek, Lynn M. Yee

**Affiliations:** ^1^ Division of Maternal–Fetal Medicine, Department of Obstetrics and Gynecology, Northwestern University Feinberg School of Medicine, Chicago, Illinois, USA.; ^2^ Division of General Internal Medicine and Geriatrics, Departments of Medicine and Preventive Medicine, Northwestern University Feinberg School of Medicine, Chicago, Illinois, USA.

**Keywords:** health care provider, health disparities, postpartum, postpartum care, provider perspective

## Abstract

***Background:*** Recent paradigm shifts in postpartum care have conceptualized the “fourth trimester” as a critical transitional period requiring tailored, ongoing health care. However, this concept presents challenges for providers, especially in low-resource settings. Our objective was to understand providers' perspectives on challenges in postpartum care to highlight strategies for optimizing care.

***Methods:*** Focus groups were conducted using a semistructured interview guide to elicit perspectives on barriers and facilitators to postpartum care. Participants included physicians, nurses, and social workers who care for low-income postpartum individuals. Interviews explored the provider experience of postpartum care, with a focus on barriers experienced by patients and providers, and tools for maintaining engagement. Analysis was performed using the constant comparative method and framed by the Social Ecological Model.

***Results:*** Participants (*N* = 26) all acknowledged the importance of the “fourth trimester” but identified multiple barriers to providing optimal postpartum care. Challenges providers perceived for patients and those they perceived for themselves often overlapped, including difficulty with appointment scheduling, insurance limitations, lack of provider continuity, and knowledge gaps. Providers identified ease of referrals to specialists, access to tangible services (*e.g.*, contraception), and enhanced care coordination (*e.g.*, patient navigation) as potential facilitators of improved postpartum care.

***Conclusions:*** Obstetric providers recognize the importance of postpartum care yet highlighted significant systems- and patient-based barriers to achieving optimal care. The development and implementation of postpartum care delivery system redesign, such as the use of patient navigators to improve health care utilization and resource attainment, may enhance care during this critical time. Clinical Trial No.: NCT03922334.

## Introduction

In 2018, the American College of Obstetricians and Gynecologists (ACOG) released guidelines for optimizing care in the immediate postpartum period (up to 12 weeks postpartum), also known as the “Fourth Trimester.”^1^ This framework describes postpartum care as an ongoing process rather than a single appointment, and highlights the importance of ensuring appropriate transitions of care.^1^ Although the concept of the Fourth Trimester is not novel—authors have been describing the “crises” of the Fourth Trimester since 1973—the challenges postpartum people face have become more stark in recent years due to rising comorbidity and an increasingly fragmented and complex health delivery system, and assuring optimal care in the postpartum period remains elusive.^2–4^

Although optimal postpartum care, or care that is comprehensive, accessible, timely, effective, and patient centered, is difficult to attain in general, it is even more challenging for individuals with low incomes.^5^ An evaluation of Medicaid-paid deliveries in Illinois during 2009–2010 found individuals who identify as non-Hispanic Black and those who were low income experienced the lowest rates of returning for a postpartum care visit.^6^ Other studies have confirmed that low income, self-identified ethnic minority status, young age, and having less than a high school education are all associated with a lower frequency of postpartum follow-up.^5,7,8^ Additionally, the content and quality of the postpartum visit and health education vary widely, with patients of low income more likely to report unmet learning needs or confusion regarding how their pregnancy impacts their future health.^9–12^ Lack of postpartum care can also lead to decreased frequency of contraception use, shorter interpregnancy intervals, and a greater risk for preterm birth.^1^ Given the well-established disparities in postpartum morbidity and the importance of pregnancy-related health to health across the life course, providing optimal and consistent care to all individuals, but especially those at greater risk, is essential to promoting optimal outcomes and lessening disparities.

Although patient factors associated with postpartum care have been explored previously, the perspectives of health care providers have not been well described.^7,8,13,14^ Understanding provider perspectives on the provision of postpartum care, particularly that provided to populations of low income, is key to improving health, increasing provider engagement, and constructing provider-led interventions in the postpartum period. Thus, we examined provider experiences and perceptions to understand the barriers and facilitators to ideal postpartum care, and the methods by which postpartum care may be enhanced.

## Methods

This was a qualitative study of focus groups of health care providers who care for individuals at Northwestern Memorial Hospital's Prentice Ambulatory Care Clinic, a comprehensive women's health practice, which provides care for individuals in an urban Chicago location. The majority of individuals receiving care in this practice have publicly funded insurance (*i.e.*, Medicaid and Medicare) for prenatal care. This practice provides care for patients who need obstetric specialist care (*N* = 47 residents and 11 faculty) as well as maternal–fetal medicine subspecialist care (*n* = 3 fellows and 14 faculty). Additional providers in the practice include a wide array of specialists, such as social workers (*n* = 2) and obstetric nurses (*n* = 4). All providers who care for patients in the postpartum period were eligible for study participation, with the final study population being a convenience sample based on schedule availability. The physician sample purposefully was weighted to include predominantly residents and fellows, based on their central role in caring for this patient population. All providers were over age 18 and were English speaking.

A semistructured interview guide was utilized during the focus groups (example topics in Table 1). Each group lasted ∼90 minutes and focused on barriers and facilitators to postpartum care for patients who receive care in this setting. Providers were queried on their perceptions of care in the early postpartum period (up to 12 weeks postpartum) as well as their perception of patients' experiences. The groups were digitally recorded and professionally transcribed without any identifying information. All were in person except the last two, which were held during the coronavirus pandemic and thus conducted using video conference technology. Each participant received a gift card. Focus groups were continued until thematic saturation was reached. The Institutional Review Board of Northwestern University approved all study activities. All participants provided written informed consent before participation.

**Table 1.** Semistructured Interview Content

Content categories	Example topics
Challenges to postpartum care	Greatest challenges to low-income pregnant and postpartum women
	Difficulties for providers, including nonclinical task burden
	Resources lacking in the provision of postpartum care
Retention in postpartum care	Indicators and motivators for follow-up postpartum
	Most effective methods to engage patients outside of the clinical setting
	Impact of social media and community groups
Current effective practices	Qualities of productive community or virtual groups
	Areas of clinical practice that currently run smoothly
	Resources currently utilized in the clinic

A secure data management and qualitative data analysis software, Dedoose, was used to facilitate analysis. This analytic process included applying the themes to the Social Ecological Model, which framed the multifactorial burdens and system limitations for both patients and providers. The Social Ecological Model describes how environmental, societal, institutional, interpersonal, and individual factors impact and influence the actions and health behaviors of individuals.^15^

Two authors (R.S.R., E.C.D.) analyzed the data using the constant comparative method, in which themes and subthemes emerged throughout the interviewing and analytic process.^16^ Analysts developed an initial codebook through an exploration of the first two transcripts. Code definitions were shared and refined with the entire research team. This codebook was then applied by each analyst to all remaining transcripts. A combined iterative process followed, allowing ineffective themes to be reclassified and discrepancies between the coders to be discussed and resolved. All transcripts and themes underwent final in-depth discussions and reevaluations to ensure agreement on the final themes. A total of 540 excerpts were coded and organized by ecological level with exemplary quotations. The shared impact of each barrier subtheme—whether providers perceived the theme to predominantly affect them versus predominantly affect patients—was also evaluated. As this study was conducted entirely with health care providers, all results are the perceptions of providers, including perceived patient-based barriers.

## Results

### Participants

From October 2019 to May 2020, interviews were conducted with 26 providers: 25 participated in 6 focus groups, and 1 was interviewed alone due to scheduling constraints (Table 2). Participants included 20 physicians, 4 obstetric registered nurses, and 2 licensed clinical social workers. Participants had been providing obstetric care for a duration ranging from 1 year (resident) to over 20 years (faculty).

**Table 2.** Provider Participants (*N* = 26)

Job category	Participant subtype	*n*
Physicians	Obstetrics–gynecology residents	15
	Maternal–fetal medicine fellows	3
	Obstetrics–gynecology specialist faculty	1
	Maternal–fetal medicine subspecialist faculty	1
Nurses	Registered nurses	4
Social workers	Obstetric licensed clinical social workers	2

**FIG. 1.**
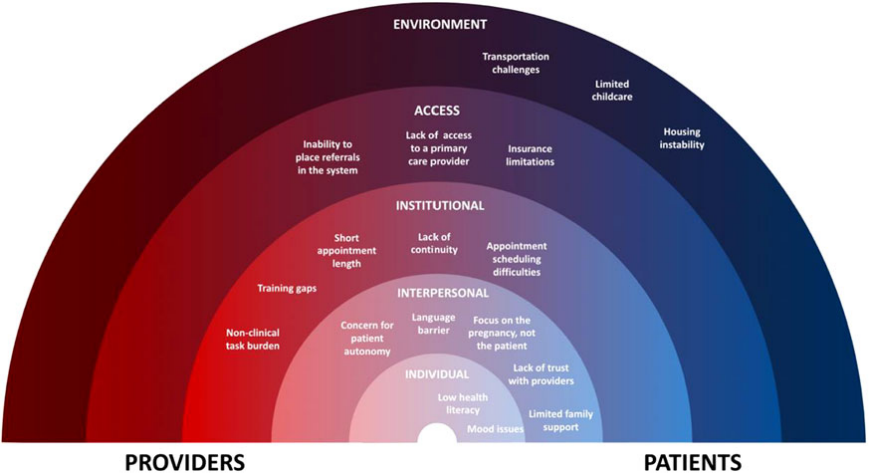
Continuum of challenges for patients and providers in the Social Ecological Model.

Participants discussed perceived challenges for providers and patients in the postpartum period (Table 3), described as barriers to postpartum care. They then reflected on facilitators of optimal care in the postpartum period (Table 4). Significant overlap between provider perceptions of provider-based versus patient-based themes was noted as well, and provider-proposed shared barriers were explored on a spectrum (Fig. 1).

**Table 3.** Provider-Perceived Barriers for Patients and Providers in the Postpartum Period

Theme	Subtheme	Exemplary quotation
Environment	Housing instability	“A lot of our patients are housing unstable and so all the support and money when you're pregnant but postpartum is really challenging.”
	Limited childcare	“So oftentimes when I have patients who don't show, when I call to see hey, are you still coming,…the most common reasons that I hear are I didn't have a way to get to the clinic or I don't have anyone to watch my kids.”
	Transportation challenges	“Being able to get downtown by car, by bus, is the weather prohibiting being able to get here by bus, is parking too costly and like prohibitive to come to a visit.”
Societal	Insurance limitations	“Patients navigating their insurance for the first time whether they are uninsured and now because of the pregnancy have become eligible to receive Medicaid and navigating that whole process can be very challenging for patients.”
	Lack of access to primary care provider	“A lot of people don't have primary care doctors. So after their one visit with us, if they still have issues, a lot of them haven't been hooked up to other types of care yet.”
Institutional	Appointment scheduling difficulties	“I'll notice that sometimes patient you know have a hard time knowing what number to call, how to make those appointments and sort of yeah, how to do those follow up things.”
	Short appointment length	“I just don't think in 15 minutes we can see an OB patient, counsel them, like take care of what they medically need and counsel them on whatever stage of pregnancy they're in, I just find it really difficult.”
	Inability to place referrals in the system	“A lot of it is sort of the responsibility is on the patient. Like we can't really get them in to see primary care at [institution]. It's really challenging.”
	Lack of continuity	“I don't have a provider that I identify as my provider, and so I don't have that like sense of trust and that willingness to engage because I'm not like, hey Doctor Smith, I saw you 12 times during my pregnancy…”
	Nonclinical task burden	“The paperwork that needs to be filled out, like FMLA paperwork and there are a lot of those kinds of tasks and trying to get like breast pumps and like all of those kind of things that I think end up being burdensome and obviously are high priority for patients.”
	Training gaps	“Even though they do teaching with us about breastfeeding and troubleshooting, I … don't have like the expertise or the experience to troubleshoot with patients.”
Interpersonal	Lack of trust with providers	“I think mistrust can play a major role and when patients don't feel that they have like a trust in their provider that we're not going to be able to provide the best care, it kinda goes both ways.”
	Language barrier	“I also think sometimes it's a language barrier. Like they pretend to know what, they pretend and they agree they do know what they're being told. But then if it's Spanish I could speak to them and then they're like, no I did not understand that.”
	Concern for patient autonomy	“We all kind of struggle with this question…is how much do you do for our patients in terms of like trying to help them and coordinate their care but also like wanting to like empower them to also…have some agency over their own healthcare.”
	Limited family support	“I think you know like the complete overwhelmed feeling that they might have after delivering and trying to manage all of that, so I think you know support from family… actually having someone there that is almost as engaged in their care as they are, I think that's a huge challenge.”
	Focusing on the pregnancy, not the patient	“I think we systemically tell women they are valued because they're pregnant and not valued because they are postpartum and the way that they are able to access care because they're able to you know get insurance and then the frequency with which we see them…”
Individual	Asymptomatic disease processes	“The fact that the acute health problem has passed and most women in the postpartum period are fine or feel fine.”
	Low health literacy	“I think one of the challenges is health literacy. They don't understand the importance of certain points in prenatal care and or postpartum, simply because they don't understand a bigger broader picture of it.”
	Postpartum mental health issues	“I think like there's some level of mood changes in every postpartum woman that is difficult to navigate.”

FMLA, Family and Medical Leave Act; OB, obstetrics.

**Table 4.** Provider-Perceived Facilitators for Improved Postpartum Care

Theme	Subtheme	Exemplary quotation
Environment	Coordinating postpartum and newborn care	“I think that would actually be pretty fantastic if that was something that we could offer our patients, is like you get baby checked, you get checked.”
Societal	Community groups	“I know there's some really good data on group prenatal classes in the community…I think that would be really helpful and increase the engagement.”
	Peer-to-peer education and support	“Just colloquially patients mentioned talking with their friends, their classmates, their family members about certain aspects of prenatal care and a lot of those conversations make a much bigger impact on their decisions to do or not do certain things than our discussions with them.”
	Social media groups	“ACOG has an Instagram page, and I know at one point they were talking about maternal morbidity and mortality… the amount of like conversations that it started, I think were very helpful and I think that they were bringing awareness about things to patients they otherwise didn't realize..”
Institutional	Access to social worker	“I think our access for a social worker is really great and she is very very on top of it about being in communication.”
	Complex antenatal courses leading to improved postpartum follow-up	“I think people engage when they have an issue but other than that they certainly are less likely to come to their postpartum visit.”
Interpersonal	Counseled on importance of follow-up	“The more we mention and communicate those things I think the more likely patients are to internalize it and then follow up postpartum or like prioritize their health postpartum as well.”
	Continuity with providers	“Patients who have been [seen] by the same provider and they've been lucky enough to get that continuity and they know that they're going to go back and see that same provider again in the future, that's an increased likelihood that they're going to return.”
	Clearer guidelines of when patients should return	“We now have specific parameters for a variety of different hypertensive situations and like exactly how long they need to follow up..”
	Telemedicine to facilitate more frequent follow-up	“I think it's something that has been really nice about this period of time is like actually being able to call a patient and feeling empowered to do that.”
Individual	Increased educational resources	“Do a standardized like discharge instruction text … that varies slightly by medical problem, so there's one for like healthy patients, there's one in Spanish, there's one for hypertensive patients and diabetic patients, like what if we just threw a resources section on the bottom of that discharge instructions template that went to every patient.”
	Tangible gains from attending appointments	“Postpartum contraception can be a big motivating factor for people to engage with their postpartum visit.”

ACOG, American College of Obstetricians and Gynecologists.

### Environmental factors

Environmental factors influencing the ability of individuals to access care were defined as factors related to the broader environment outside of the health care setting. Subthemes of environmental barriers included housing instability, limited childcare, and transportation challenges (Table 3). Childcare, not only for the neonate but also for other children at home, was a frequent concern for patients, resulting in missed appointments. Transportation considerations included inaccessibility and cost of public transportation, cost of parking, and distance to travel for frequent appointments. It also encompassed the inability to access time away from work to attend appointments. One participant summarized these environmental barriers as: “Oftentimes when I have patients who don't show…the most common reasons that I hear are I didn't have a way to get to the clinic or I didn't have anyone to watch my kids.”

Only one environmental facilitator of postpartum care was identified (Table 4). Specifically, many providers recommended coordinating postpartum and newborn care as an effective way to overcome some of these barriers. One participant said: “I think something that could be done to help facilitate that is like if we could…coordinate even with like the baby's care so that if the mom was gonna come to the pediatrician's visit, like we could do the blood pressure check that day.” The concept of a family-centered medical home was suggested as an example of this type of care.

### Societal factors

Societal factors related to a patient's ability to access optimal health care at an affordable cost as well as the influence of groups in their community on their antepartum and postpartum experience. Subthemes included insurance limitations and lack of access to a primary care provider. The complexity of obtaining and maintaining insurance was identified as an important barrier. Providers highlighted the importance of primary care, stating “I try to make sure that the most important thing they hear from me is that they need to have a primary care doctor,” although participants commonly expressed frustration that access was limited by insurance coverage.

Providers identified community groups, peer-to-peer education and support, and social media groups as facilitators of enhanced care in the postpartum period (Table 4). Community groups (*i.e.*, prenatal courses in the community) were viewed as ways to effectively bring health-related knowledge to patients and improve postpartum health. Providers noted that patient conversations with family or friends resulted in “a much bigger impact on their decisions to do or not do certain things than our discussion with them.” Providers saw a benefit of social media groups for patient education and emotional support, especially for those with medical comorbidities: “I've seen it for patients who have fetal anomalies who will join support groups and they'll talk about their antepartum course and their postpartum course with their child that is affected by X, Y, Z birth defect.” However, providers warn about the risks of misinformation from social media:

“Social media is wonderful, there's so many peer benefits for peer support, but when they're not monitored that's when…the misinformation, the information that's wrong comes in about contraception, postpartum depression, baby moving means that they're in distress.”

### Institutional factors

Institutional issues addressed barriers or facilitators to effective patient care within the hospital system and clinic environment. Providers described appointment scheduling difficulties, short appointment length, inability to place referrals within the system, lack of continuity, nonclinical task burden, and training gaps as notable barriers (Table 3). In expressing scheduling issues, their patients face, one provider stated: “Sometimes patients you know have a hard time knowing what number to call, how to make those appointments and…how to do those follow-up things.” The notion that even when patients could access care, the appointment time was insufficient also surfaced repeatedly. Additionally, many voiced frustrations with the inability to link a patient into primary care within the same institution, given constraints related to practices accepting public insurance: “Here at [institution] we can't refer them to internal or family medicine because they don't accept the insurance and so I think that's also a barrier.”

Another central theme in all focus groups was the lack of continuity in a group obstetric practice. One clinician provided this example from a patient's point of view (“I don't have a provider that I identify as my provider, and so I don't have that like sense of trust and that willingness to engage”) and the provider point of view (“There's only so much that the longitudinal problem list can do to account for conversations that really need to be ongoing throughout the course of prenatal or postpartum care”). Additional concerns on the provider side included the nonclinical task burden as well as concerns regarding provider training limitations, including a lack of focus on certain postpartum issues (Table 3). Participants noted a large nonclinical task burden, such as completing paperwork to access resources or writing letters to help their patients achieve flexibility at work, as well as taking on a care coordination and resource attainment role.

Two pathways that facilitated patients' return to postpartum care were noted throughout the focus groups. The first was access to social workers, who can assist with facilitation of resource attainment and mental health care provision (Table 4). Providers identified that some of the institutional barriers, such as scheduling and lack of continuity, may be ameliorated by the inclusion of additional health care team members, such as patient navigators, who focused on supporting these tasks. Providers also identified that complex antenatal conditions led to improved postpartum follow-up at this center is a facilitator (Table 4): “I think specifically for our…very sick patients that have multiple subspecialists following them….I think they are probably more likely to follow up because they have so many important appointments.” The concept that patients with added medical complexity may have received better care and health education than patients without such complexity was acknowledged by providers who stated that low-risk patients may benefit from similar care coordination.

### Interpersonal factors

Interpersonal dynamics were described as those for which health care use and outcomes were directly influenced by people. Interpersonal barriers centered on patient relationships and included lack of trust with providers, language barriers, concern for patient autonomy, limited family support, and a focus on the pregnancy rather than the patient (Table 3). Mistrust in unfamiliar providers along with mistrust of the medical system at large, as well as language barriers were major concerns. The need to balance providers' facilitation of care with patient autonomy was a theme central to this discussion. Providers reported a tension in which the concern was not that they could not do more, but that they should not do more for patients in terms of care coordination and follow-up (Table 3). One provider described the tension as:

“I also personally struggle with the push and pull between giving patients their autonomy as they're adults who are taking care of kids and have their own schedules to deal with, and then us making appointments for them…I don't know whether that's actually helping or not.”

Patient-centered interpersonal barriers to postpartum care included limited family support, with providers stating concerns such as, “it's hard to find the family and support who are able to help.” Finally, the belief that patient needs are not taken as seriously in the postpartum period was expressed multiple times. One provider, when portraying this barrier from the patient's point of view, stated, “This was like super heartbreaking, that the doctors seemed to care about me so much when I was pregnant and all these things were so important…but then I had my baby and then all of a sudden none of those things were important anymore.”

Multiple interventions to improve care at the interpersonal level were identified (Table 4). These included counseling on the importance of follow-up, continuity with providers, clearer guidelines of when patients should return, and telemedicine to facilitate more frequent follow-up. Providers expressed that having clearer guidelines for both patients and providers on when to follow-up, and why it is important was a major emphasis of conversations on how to improve postpartum care. Additionally, the ability to have continuity with providers was noted to be essential in building trust and rapport with patients. Another major facilitator of optimal care postpartum was utilizing available electronic medical records for interprovider consistency and communication, as well as patient ease of communication through the electronic medical record's portal function. The final two focus groups, which took place after coronavirus disease 2019 (COVID-19) had emerged, lent themselves particularly to discussions of telemedicine. These conversations often centered around the idea of virtual medicine as a tool for increasing care retention, lessening the burden of travel and childcare, and allowing for more frequent communication. In describing telehealth, one provider stated it “is huge actually…an ability to access a doctor and troubleshoot postpartum issues over the phone in a formal way…would be a huge win for all moms.”

### Individual factors

Individual concerns were defined as issues related to patient and providers directly without influence of other people or institutions. Individual-level barriers that providers ascribed to patients included asymptomatic disease processes, low health literacy, and postpartum mental health issues (Table 3). Many providers expressed frustration at patients who did not follow-up due to “feeling fine,” one stating that “I definitely had a patient come in after six weeks for a complaint who was like…I was feeling healthy at six weeks, so I didn't come in.” Regarding low health literacy, providers often cited a lack of patient understanding of health conditions. For example, one physician described a patient who did not understand her preeclampsia diagnosis and endorsed a lack of “understanding what that even means, what the name of the disease is and what the symptoms associated are.” Postpartum mental health issues, most notably postpartum depression, were also perceived as a factor that limited patient engagement in care (Table 3).

Facilitators for improving postpartum care included increased educational resources and making clear the tangible gains from attending appointments (Table 4). Examples of informational resources included handouts, discharge instructions, websites, or other information from health care team members. Tangible gains included contraception, blood pressure checks, or participation in a peripartum mood disorder program offered at this clinical site. One provider aptly described their belief about why patients return to postpartum care:

“A lot of times patients just come back because they…want an IUD or they need like a contraception plan if they're engaged in care but otherwise [without those tangible benefits] I think…they…feel like they're healthy enough and now their focus is the baby.”

## Discussion

The recent professional and public health commitment to the “Fourth Trimester” highlights the importance of effective and longitudinal postpartum care. However, many challenges to optimal postpartum care exist. In this analysis of provider perspectives on the delivery of postpartum care, barriers to care were identified to exist at every level of the Social Ecological Model.^15^ These barriers were myriad, and included factors as broad as childcare and health insurance issues, and as focused as individual provider knowledge, provider continuity, the need for resource coordination support, and scheduling challenges. However, facilitators were also identified at each level, suggesting provider input may be one key to improving some of the fractures in postpartum care.

Findings from prior research are consistent with many of the findings in this study. A qualitative analysis of postpartum care among clinicians in New York City noted similar concerns over lack of training in psychosocial and resource attainment issues, as well as lack of continuity of postpartum care.^17^ Insights derived from patients in prior studies mirror many of the concerns providers expressed in our study. One study in which postpartum individuals were enrolled found the most commonly cited reason for nonadherence to follow-up was “feeling fine.”^7^ Similarly, in another analysis of patients' postpartum concerns, commonly cited themes included a need for social support, desire for education on issues such as breastfeeding and newborn care, and insurance concerns.^18^

The present analysis revealed that providers perceive a significant burden on themselves as clinicians as well as on their patients to ensure effective and accessible postpartum care for individuals with fewer socioeconomic resources. Many systems issues were identified, including insurance barriers to specialist and primary care within the same health system, issues with appointment making and keeping, and lack of provider continuity that negatively impacted both patients and providers (Fig. 1). Our findings suggest adequate postpartum care for all individuals will require societal and environmental changes, such as access to transportation, childcare, providers in network, or universal insurance. These changes require significant efforts on the policy side, such as recent efforts to expand Medicaid coverage to 1 year postpartum.^19^ However, change is also required on a local level, as providers identified areas in which both providers and patients require additional support. This support may come in the form of administrative assistance, structural changes in clinic flow, support for care coordination, and increased access to educational resources for all parties.

Despite the medical and psychosocial importance of the postpartum period, the routine achievement of comprehensive and longitudinal postpartum care has not yet become a national priority. While Healthy People 2020 had the goal of increasing the proportion of individuals who have a postpartum visit, Healthy People 2030 only asks for adequate screening of postpartum depression.^5,20,21^ Additionally, the lack of nationwide data on postpartum health has resulted in difficulty in assessment of the quality of postpartum care.^21^ Thus, further research on postpartum quality improvement is critical to innovate and redesign care. Our findings highlight the need to include provider perspectives in addition to those of patients and other stakeholders in such processes.

Several programs rooted in both health behavior modification and resource allocation have already demonstrated methods by which postpartum care may be redesigned.^14,22,23^ For example, a program that included home visitation and peer support decreased postpartum depression and improved maternal satisfaction with services offered.^22^ Additionally, peripartum educational programs may improve effective contraception use and decrease unplanned pregnancies.^22^ With regard to financial barriers, a program in New York City found an educational component matched with financial assistance resulted in increased attendance at scheduled postpartum visits and an increased likelihood to remain enrolled in their Medicaid plan at 6 months and 1 year after delivery.^14^ Postpartum patient navigators have been suggested to promote self-efficacy through providing education, shared decision making, emotional support, and support for reducing barriers to health care access.^24^ The present analysis, which was conducted in preparation for a randomized trial of a yearlong postpartum patient navigation intervention, suggests that redesigning postpartum care to include additional members of the health care team to ease the burdens of both patients and providers may be one way to optimize care for all.

While this study was able to provide insight into the breadth of opinions among several types of health care providers, it was limited to providers at one urban, tertiary academic medical center. Additional work would be required to understand the perspectives of providers in other settings, geographic locations, and care delivery systems. Incorporating the perspective of health system administrators and other health care team members may offer further insights on barriers and potential solutions. Barriers and facilitators may also vary by patient population; for example, prior qualitative work on postpartum care for immigrant individuals found provider concerns about the patient experience of cultural stigma and culturally incompetent care, which were themes that did not arise during our focus groups.^25^ Finally, provider perceptions of patient barriers as expressed in this study are not necessarily reflective of actual patient barriers, and do not account for provider implicit biases that may influence patient health. For example, prior qualitative research on the low-income minority patient experience in the postpartum period found that a third of patients perceived coercion or racially based discrimination in contraception counseling.^26^ Having a greater understanding of patient perceptions of challenges, including relationships with providers, is another critical part of understanding the postpartum period, which this study did not aim to address.

## Conclusion

In the context of a renewed commitment to optimal postpartum care, this study explores the perceptions of providers in an urban setting caring for low-income individuals. The barriers and facilitators identified by the providers elucidate major roadblocks as well as proposed paths to achieve this goal. Providers and patients both face immense challenges at all ecological levels that limit their ability to achieve optimal care in the postpartum period. Redesigning postpartum care to account for these issues may ultimately result in higher quality, compassionate care for all postpartum individuals, regardless of the socioeconomic status.

## Disclaimer

The content is solely the responsibility of the authors and does not necessarily represent the official views of the National Institutes of Health.

## Abbreviations Used

ACOG: American College of Obstetricians and Gynecologists
COVID-19: coronavirus disease 2019
FMLA: Family and Medical Leave Act
OB: obstetrics
